# Repeatability and agreement of AOCT-1000 M, RTVue XR and IOL master 500 in measuring corneal thickness mapping and axial length applying principle of optical coherence tomography

**DOI:** 10.1186/s12880-023-01147-7

**Published:** 2023-11-21

**Authors:** Hailong Ni, Suzhong Xu, Li Tian, Jieli Mao, Jing Li, Na Lin, Peike Hu, Zhiyi Wu, Xiang Chen, Zhishu Bao, Jingwei Zheng, Peihua Yan, Ruzhi Deng

**Affiliations:** 1 Eye Center, Second Affiliated Hospital, School of Medicine, Zhejiang University, Hangzhou, 310010 China; 2 Eye hospital of Wenzhou Medical University, Wenzhou, Zhejiang 32500 China; 3 Vision X Medical Technology Co., Ltd., Shanghai, 201112 China

**Keywords:** Repeatability, Agreement, Central corneal thickness, Axial length, RTVue XR, AOCT-1000 M

## Abstract

**Purpose:**

To evaluate the repeatability and agreement of Fourier-domain optical coherence tomography (AOCT-1000 M and RTVue XR) and partial coherence interferometry biometer (IOL Master 500) in measuring corneal thickness mapping and axial length respectively.

**Methods:**

Corneal thickness was measured by AOCT-1000 M and RTVue XR. Axial lengths were measured by AOCT-1000 M and IOL Master 500. The repeatability and agreement of corneal thickness and axial length were calculated in two groups of devices. The intraclass correlation coefficient (ICC) was used to verify the repeatability of the device. The 95% confidence interval of the difference compared to the set cut-off value was used to verify the agreement between the two devices.

**Results:**

A total of 60 subjects with 58 eyes were included. The central corneal thickness measured by AOCT-1000 M and RTVue XR were 504.46 ± 42.53 μm and 504.43 ± 42.89 μm respectively. The average difference between groups was 0.03 ± 4.58 μm, and the 95% confidence interval was (-1.17, 1.24), which was far less than the set threshold value of 15 μm (P < 0.001). Both RTVue XR and AOCT-1000 M had very good ICC values of central corneal thickness (0.998 and 0.994, respectively). The average axial lengths measured by AOCT-1000 M and IOL Master 500 were 24.28 ± 1.25 mm and 24.29 ± 1.26 mm respectively and the 95% confidence interval was (-0.02, 0.01), which was less than the set threshold value of 0.15 mm (P < 0.001). The ICC for both devices were 1.000.

**Conclusion:**

Good repeatability and agreement were seen in measurements of central corneal thickness and axial length by AOCT-1000 M.

## Introduction

Myopia is increasing worldwide, and is estimated to affect nearly 4758 million people globally by the year 2050 [1]. The onset and progression of myopia are related to the interaction between environmental and genetic factors [2], which disturbs the normal eye growth, leading to an increase in axial length. Prevention and control of myopia is critical because the rate of myopia-associated complications significantly increases in high myopia with extremely long axial length [3]. Refractive surgery [4] is a popular method of vision correction in myopic patients. However, keratoconus, which develops from corneal thinning, is an absolute contraindication for corneal refractive surgery [5], and its detection is very important in refractive surgery screening [6, 7]. Therefore, accurate measurement of corneal thickness mapping and axial length are very important in refractive surgery screening and myopia progression monitoring.

The epidemiology of myopia has led to the development of biometers by some manufacturers, which are suitable for optometry centers or grassroots hospitals, and can measure the biometry parameters including corneal thickness mapping and axial length for refractive surgery screening and myopia progression monitoring. One such instrument is the AOCT-1000 M (Hangzhou Weixiao Medical Technology Co., Ltd.), which uses the optical coherence tomography(OCT) technique to measure the corneal thickness mapping and determine the axial length, at a low cost.

RTVue XR is a clinically-approved OCT instrument, which can measure the corneal thickness mapping with an adapter for cornea imaging. The central corneal thickness measurement of RTVue XR has good repeatability [8, 9].

The IOL Master 500 is a fast optical biometer that is based on partial coherence interferometry (PCI), and has been commonly used to measure ocular parameters for several years [10]. The measurement results are accurate and reliable with high measurement repeatability of axial length [11, 12]. Neither the clinical performance in terms of repeatability of the AOCT-1000 M nor its agreement has been assessed with the clinically approved techniques in normal and diseased eyes. Therefore, the purpose of this study was to assess the repeatability of AOCT-1000 M and its agreement with RTVue XR in measuring the corneal thickness mapping and with IOL Master 500 in measuring the axial length in subjects with normal and diseased eyes.

## Data and methods

### General information

This study is a prospective, multi-center and self-controlled clinical evaluation. Patients who were admitted to the Eye Hospital of Wenzhou Medical University and Eye Center, Second Affiliated Hospital of Zhejiang University from March to June, 2022. All the patients with age more than 18 years are selected in this study. We have excluded the individuals having fundus diseases that may affect the examination of the axial length, non-contact intraocular pressure higher than 21 mmHg, who have received photodynamic therapy, obvious photophobia, who wear contact lenses on the day of examination, active eye diseases, participated in other clinical research within one month before being selected, and those who are pregnant, lactating or planning to become pregnant in the near future. This study has passed the ethical examination and approval of both centers, and all subjects have understood the purpose and significance of this study. They have also signed the informed consent form before being included in this study.

### Methods

**Fig. 1:**
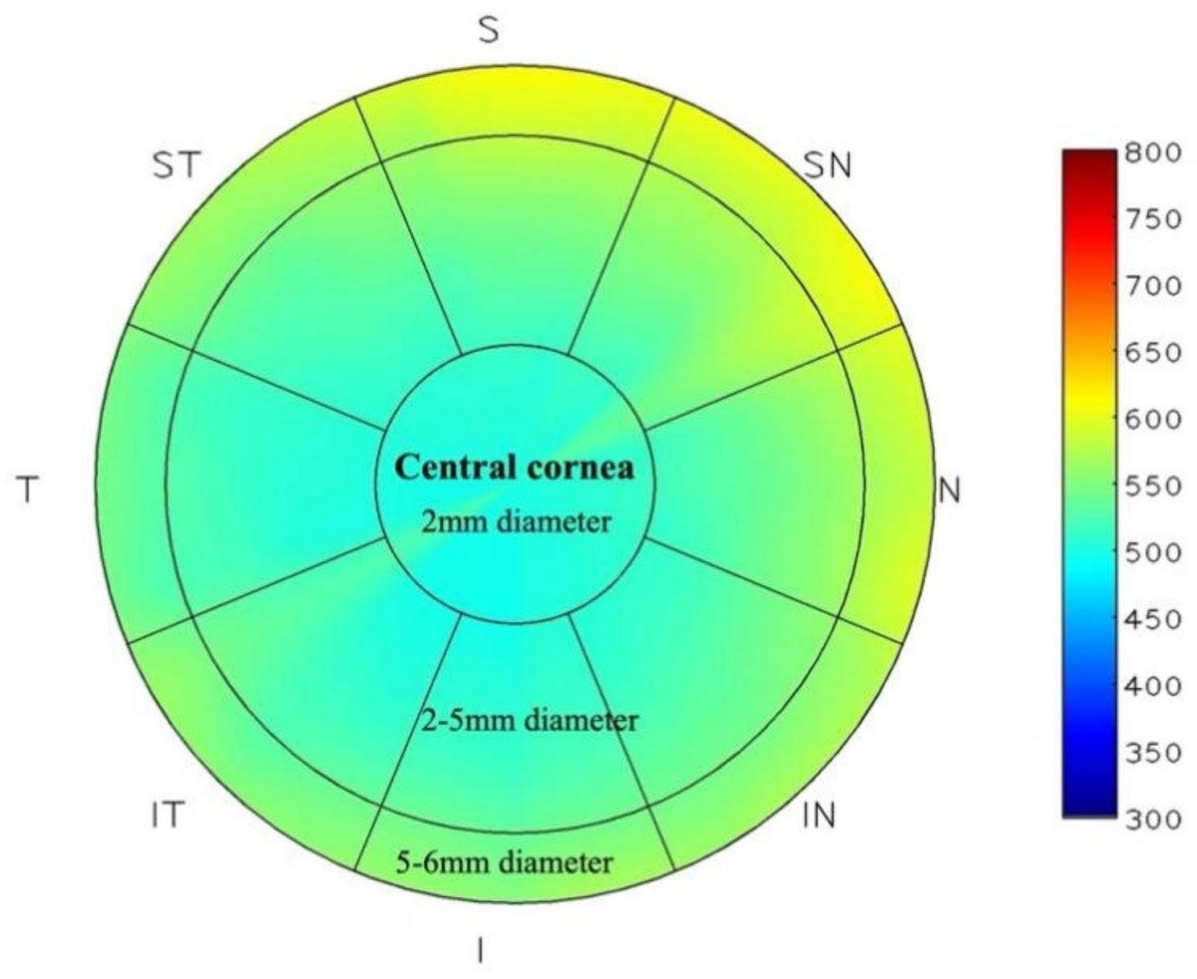
The central (2 mm diameter area) and peripheral (2–5 mm and 5–6 mm diameter area) corneal thickness were measured by two devices. Superior, supranasal, nasal, infranasal, inferior, infratemporal, temporal and supratemporal were abbreviated by S, SN, N, IN, I, IT, T and ST, respectively

The AOCT-1000 M, developed by Hangzhou Weixiao Medical Technology Co., Ltd., and the RTVue-XR, manufactured by Optovue Inc. in Fremont, CA, USA, employ spectral domain OCT technology. The IOL Master 500, produced by Carl Zeiss Meditec in Jena, Germany, utilizes partial coherence interferometry (PCI),which measures the axial length of the eye. The AOCT-1000 M was used as experimental device and the RTVue-XR was used as control device to measure the central corneal thickness (2 mm diameter) and peripheral corneal thickness (2-5 mm and 5-6 mm diameter area) which divided into 8 directions (superior, supranasal, nasal, infranasal, inferior, infratemporal, temporal, supratemporal), as shown in Fig. 1. The AOCT-1000 M was used as experimental device and the IOL master 500 was used as control device to measure axial length.

All the subjects were examined by experimental equipment and control equipments. The inspection order of the equipment was random, and each equipment was continuously used for 3 measurements with a time interval of 15s, during which patients were encouraged to blink normally. All inspections were done by a skilled technician.

### Evaluation indicators

Previous studies have found that as farther one goes to the periphery, the greater difference in corneal measurement is observed [13, 14]. Therefore, in this research project, the main indexes of agreement evaluation were central corneal thickness and axial length. The repeatability, peripheral corneal thickness of 2–5 mm and corneal thickness of 5–6 mm measured by two types of equipment were also analyzed.

### Statistical analysis

#### Calculation of sample size

It has been well-documented that the absolute value of difference between the axial length measured by IOL Master 500 and other measuring instruments is 0.002 ~ 0.2 mm [15–18]. In this study, it was assumed that the absolute value of the difference between the axial length measured by the experimental device and the axial length measured by the control device is 0.08 mm, and the standard deviation is 0.1 mm. Considering the clinical significance and acceptable clinical standards, the equivalent boundary value was set at 0.15 mm. The one-sided test level α was 0.05, and the confidence (1-β) = 90%. According to the paired design, 19 eyes needed to be included, and assuming 15%, deciduous rate actually 23 eyes needed to be included. The absolute difference between the central corneal thickness measured by RTVue XR and other measuring instruments was 0.05 ~ 17.59 μm [19, 20]. Furthermore, it was supposed that the absolute value of the difference between the central corneal thickness measured by the experimental device and the central corneal thickness measured by the control device was 10 μm, and the standard deviation was 12 μm. Taking into account the clinical significance and acceptable clinical standards, the equivalent boundary value was set at 15 μm. The one-sided test level α was 0.05, and the confidence (1-β) = 90%. According to the paired design, it was calculated by PASS16 software, where 51 eyes needed to be included. Considering the deciduous rate of 15%, 60 eyes actually were included.

Benefitting from applicable population and scope of the test instruments, the subjects were included in the following five categories: normal eyes population with no other abnormalities except ametropia, abnormal corneal morphology, corneal refractive surgery, cataract and cataract surgery. To sum up, at least 60 eyes of 60 subjects were included in this study, having at least 12 people in each of the five groups.

#### Statistical analysis

PASS16 software was used for statistical analysis. All statistical analysis tests adopt two-sided hypothesis test, and the level of hypothesis test is α = 0.05, that is, a p value less than 0.05 will be regarded as statistically significant. The distribution of the datasets was checked for normality using the Shapiro-Wilk tests, which indicated that the data were normally distributed (P>0.05).

## Results

### Demographic distribution

A total of 60 subjects participated in this study, of which one failed to obtain valid data, one dropped out of the study, and eventually 58 subjects were actually included in the analysis. The number of subjects in the two centers was 30 and 28 respectively. The age of the subjects was 41.64 ± 18.07 years, and the analysis eyes were 58. The details were shown in Table 1.

**Table 1:** Basic information of subjects

Classification	Number of subjects	
Gender	Man	22
	Woman	36
Laterality of the eye	right eye	49
	left eye	9
Type of the groups	Normal eyes except ametropia	12
	Eyes with abnormal corneal morphology	12
	Eyes after corneal refractive surgery	11
	Cataract eyes	11
	Eyes after cataract surgery	12

### Repeatability and agreement of corneal thickness mapping measured by AOCT-1000 M and RTVue XR

#### AOCT-1000 M and RTVue XR had good repeatability in measuring corneal thickness

The central corneal thickness was measured by AOCT-1000 M, which showed intraclass correlation coefficient (ICC) 0.994, and the 95% confidence interval (0.991, 0.997). Meanwhile, the central corneal thickness was measured by RTVue XR, which depicted the corresponding ICC 0.998, and the 95% confidence interval (0.997, 0.999). The ICC decreased slightly toward the periphery, but all of them were higher than 0.75 as shown in Table 2.

**Table 2:** ICC measured by AOCT-1000 M and RTVue XR in various areas of cornea

	AOCT-1000 M		RTVue XR	
Areas of cornea	ICC	95%CI	ICC	95% CI
Central cornea	0.994	(0.991,0.997)	0.998	(0.997,0.999)
**Peripheral corneal 2–5 mm diameter area**				
S	0.957	(0.935,0.973)	0.982	(0.973,0.989)
SN	0.944	(0.914,0.964)	0.974	(0.960,0.984)
N	0.950	(0.923,0.968)	0.981	(0.971,0.988)
IN	0.946	(0.918,0.966)	0.972	(0.958,0.983)
I	0.965	(0.946,0.978)	0.976	(0.964,0.985)
IT	0.963	(0.944,0.977)	0.980	(0.970,0.988)
T	0.966	(0.947,0.979)	0.990	(0.984,0.993)
ST	0.962	(0.94,0.976)	0.987	(0.981,0.992)
**Peripheral corneal 5–6 mm diameter area**				
S	0.905	(0.856,0.940)	0.896	(0.844,0.933)
SN	0.801	(0.707,0.872)	0.840	(0.767,0.896)
N	0.843	(0.771,0.898)	0.909	(0.864,0.942)
IN	0.844	(0.772,0.899)	0.868	(0.805,0.915)
I	0.864	(0.799,0.912)	0.871	(0.810,0.917)
IT	0.828	(0.749,0.889)	0.884	(0.828,0.925)
T	0.831	(0.753,0.890)	0.961	(0.941,0.975)
ST	0.875	(0.813,0.921)	0.930	(0.894,0.955)

#### Agreement of central corneal thickness

**Fig. 2:**
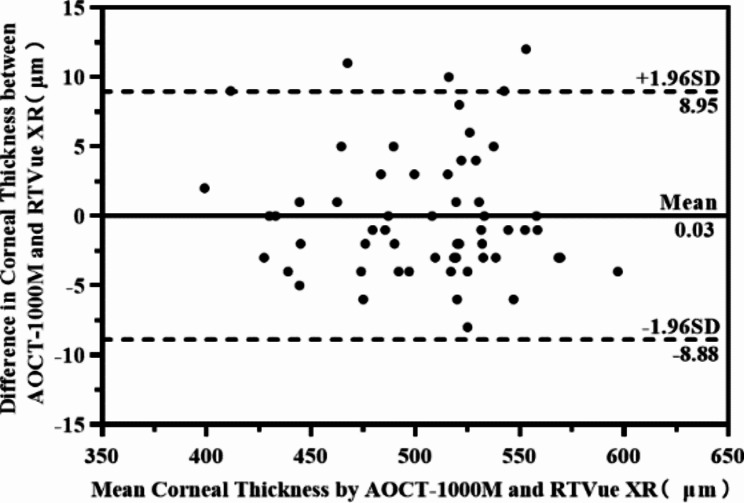
Bland-Altman plot of central corneal thickness measurements between AOCT-1000 M and RTVue XR(n = 58)

The average central corneal thickness of 58 eyes measured by AOCT-1000 M and RTVue XR was 504.46 ± 42.53 μm and 504.43 ± 42.89 μm respectively. The average difference between groups was 0.03 ± 4.58 μm, and the 95% confidence interval was (-1.17, 1.24), which was far less than the set threshold value of 15 μm (p < 0.001). The average difference between AOCT-1000 M and RTVue XR measurement for five types of eyes was 3.00 ± 5.68 μm, -2.53 ± 2.90 μm, 1.42 ± 3.62 μm, 0.64 ± 4.87 μm and − 2.19 ± 3.20 μm respectively, as shown in Table 3. Bland-Altman plotshowed that the 95% Limits of Agreement(LOA) of central corneal thickness was (-8.88, 8.955) μm, as shown in Fig. 2.

**Table 3:** Comparison of consistency between AOCT-1000 M and RTVue XR in measuring central corneal thickness in different groups

Central corneal thickness(Mean ± SD )			
		AOCT-1000 M	RTVue XR
Groups	Normal eyes except ametropia	528.53 ± 26.95	525.53 ± 27.53
	Eyes with abnormal corneal morphology	472.97 ± 34.76	475.50 ± 35.29
	Eyes after corneal refractive surgery	467.82 ± 46.66	466.39 ± 48.33
	Cataract eyes	527.70 ± 18.20	527.06 ± 19.42
	Eyes after cataract surgery	524.17 ± 33.72	526.36 ± 34.38
Statistical analysis	Mean of all groups	504.46 ± 42.53	504.43 ± 42.869
	Mean of the difference and 95% CI	0.03 ± 4.58 (-1.17-1.24)	
	Cutoff value VS. 95% CI	Difference VS. -15	Difference VS. 15
	T value	24.98	-24.87
	P value	< 0.001	< 0.001

There was difference in the central corneal thickness of different types of eyes. The central corneal thickness of eyes with abnormal corneal morphology and eyes after corneal refractive surgery were significantly lower than that of other three types of subjects without corneal abnormality (RTVue XR: F = 9.95,P < 0.001; AOCT-1000 M:F = 9.10,P < 0.001).

#### The differences of eight directions in 2–5 mm diameter areas corneal thickness between two devices

**Fig. 3:**
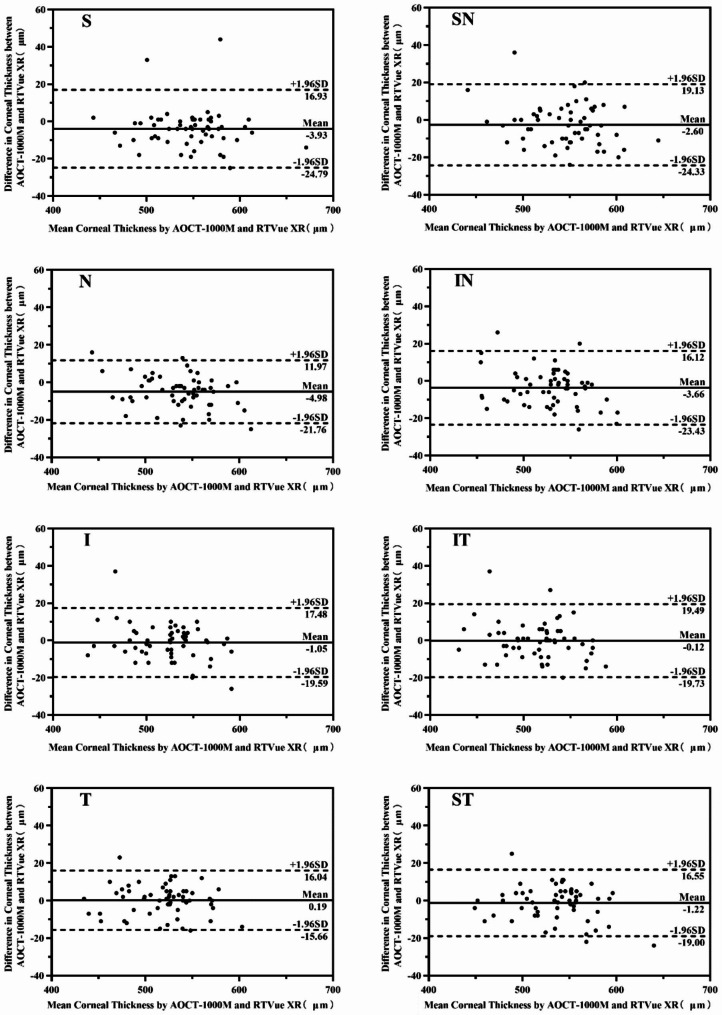
Bland–Altman plots of corneal thickness in 2-5 mm diameter areas between AOCT-1000 M and RTVue XR. S, SN, N, IN, I, IT, T and ST represented 8 directions

The corneal thickness of 2–5 mm diameter measured by AOCT-1000 M and RTVue XR in eight directions were: shown in Table 4. The 95% LOA of eight directions measured by Bland-Altman diagram was − 24.79 ~ 16.93 μm, -24.33 ~ 19.13 μm, -21.76 ~ 11.97 μm, -23.43 ~ 16.12 μm, -19.59 ~ 17.48 μm, -19.73 ~ 19.49 μm, -15.66 ~ 16.04 μm, -19.00 ~ 16.55 μm respectively as shown in Fig. 3.

**Table 4:** Differences in corneal thickness measured by AOCT-1000 M and RTVue XR in 8 directions of 2-5 mm diameter area

2–5 mm diameter area	Corneal thickness(mean ± SD)		Mean difference of two devices	95%CI of the intergroup difference
	AOCT-1000 M	RTVue XR		
S	543.81 ± 40.33	547.74 ± 41.19	-3.89 ± 10.67	-6.70, -1.09
SN	544.57 ± 38.12	547.14 ± 40.44	-2.57 ± 11.15	-5.51, 0.36
N	533.18 ± 35.84	538.16 ± 38.48	-4.97 ± 8.53	-7.21, -2.73
IN	526.17 ± 35.07	529.82 ± 37.68	-3.66 ± 10.10	-6.31, -1.00
I	521.98 ± 35.19	522.99 ± 37.81	-1.01 ± 9.45	-3.50, 1.47
IT	515.58 ± 35.25	515.66 ± 37.13	-0.07 ± 9.98	-2.70, 2.55
T	518.20 ± 36.39	518.07 ± 37.04	0.12 ± 8.00	-1.98, 2.23
ST	533.56 ± 37.66	534.87 ± 39.48	-1.31 ± 9.04	-3.69, 1.07

#### The differences of each area in 5–6 mm corneal thickness between two devices

**Fig. 4:**
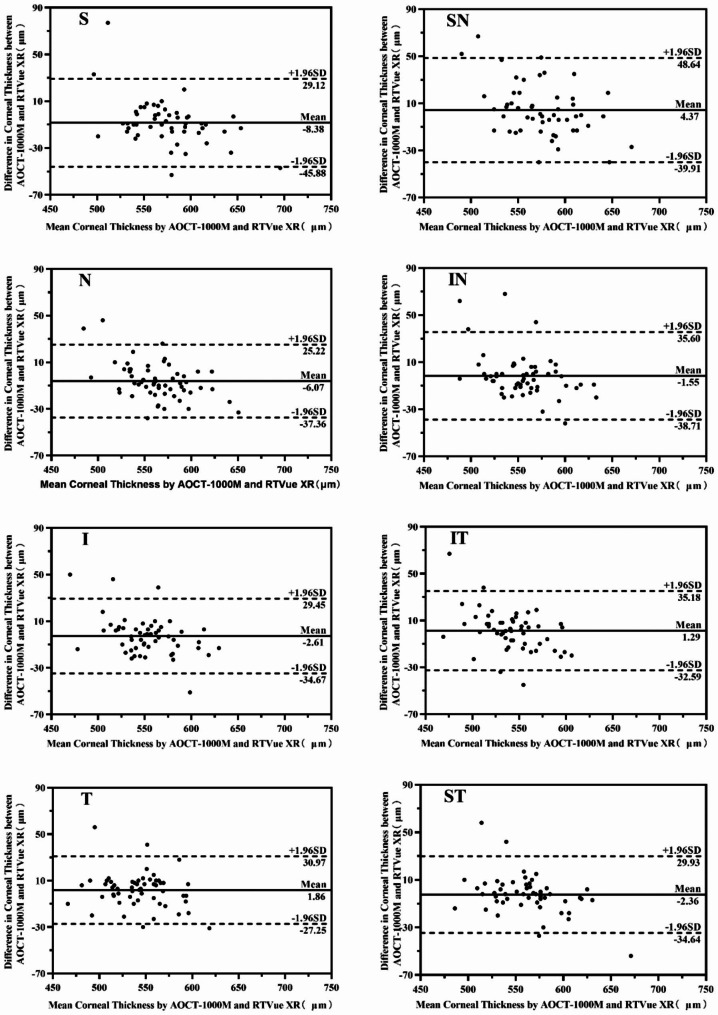
Bland–Altman plots of corneal thickness in 5–6 mm diameter areas between AOCT-1000 M and RTVue XR. S, SN, N, IN, I, IT, T and ST represented 8 directions

The corneal thickness of 5–6 mm diameter measured by AOCT-1000 M and RTVue XR in eight directions were shown in Table 5. The 95% LOA of eight directions measured by Bland-Altman diagram was − 45.88 ~ 29.12 μm, -39.91 ~ 48.64 μm, -37.36 ~ 25.22 μm, -38.71 ~ 35.60 μm, -34.67 ~ 29.45 μm, -32.59 ~ 35.18 μm, -27.25 ~ 30.97 μm, -34.64 ~ 29.93 μm respectively as shown in Fig. 4.

**Table 5:** Differences in corneal thickness measured by AOCT-1000 M and RTVue XR in 8 directions of 5–6 mm area

5–6 mm diameter area	Corneal thickness(mean ± SD)		Mean difference of two devices	95%CI of the intergroup difference
	AOCT-1000 M	RTVue XR		
S	571.63 ± 35.61	579.96 ± 43.97	-8.33 ± 19.17	-13.62, -3.05
SN	577.20 ± 34.16	572.79 ± 43.55	4.40 ± 22.64	-1.90, 10.71
N	560.88 ± 30.40	566.94 ± 37.80	-6.06 ± 16.02	-10.31, 1.81
IN	554.90 ± 30.46	556.48 ± 37.48	-1.57 ± 18.96	-6.65,3.51
I	551.29 ± 29.91	553.93 ± 36.40	-2.64 ± 16.39	-7.03, 1.75
IT	542.18 ± 27.87	540.86 ± 35.16	1.32 ± 17.25	-3.35, 5.98
T	543.88 ± 31.23	542.05 ± 34.01	1.82 ± 14.86	-2.12, 5.77
ST	560.77 ± 33.30	563.11 ± 40.26	-2.34 ± 15.54	-6.90, 2.22

### Repeatability and agreement between AOCT-1000 M and IOL Master 500 for measuring axial length

#### Repeatability of axial length

The experimental and the control equipment had extremely high ICC for the measurement of axial length, which was 1.000 in both groups. It showed that both devices had good repeatability.

#### Agreement of axial length

**Fig. 5:**
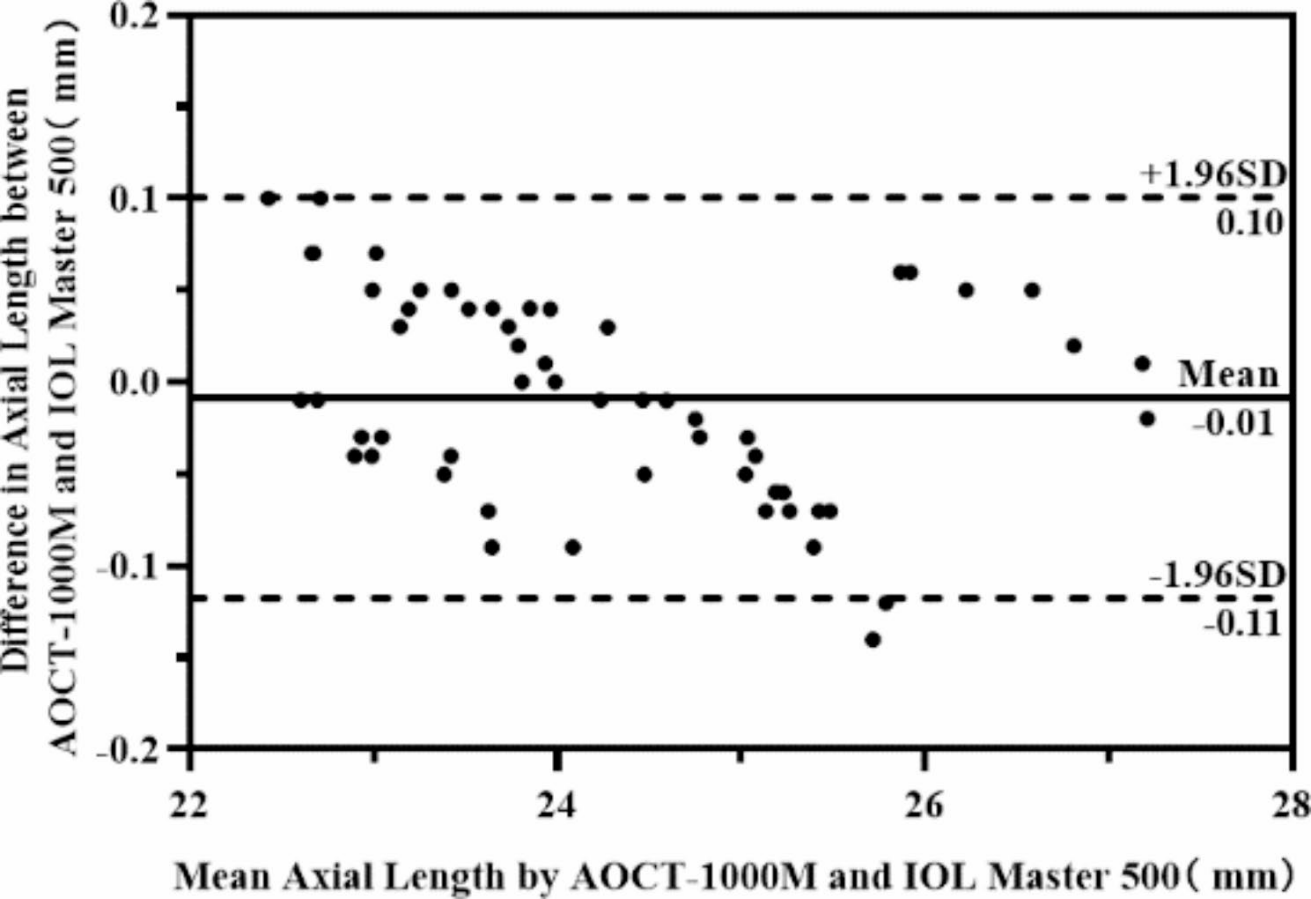
Bland-Altman plot of axial length measurements between AOCT-1000 M and IOL Master 500(n = 58)

The average axial length measured by the experimental device and the control device was 24.28 ± 1.25 mm and 24.29 ± 1.26 mm. The axial length in different groups were shown in Table 6. The average measurement difference between the two devices in 58 eyes was − 0.01 mm, and the 95% confidence interval was between (-0.02, -0.01), which was significantly less than the boundary value of 0.15 mm as shown in Table 6. The test result of Bland Altman showed that the 95% LOA of axial length was (-0.11, 0.10) mm, which was also within the set limit value, thereby indicating good agreement between the two instruments as shown in Fig. 5.

**Table 6:** Axial length measurement and agreement between AOCT-1000 M and IOL Master 500

		Axial length	
	Group	AOCT-1000 M	IOL Master 500
Axial length	Normal eyes except ametropia	25.01 ± 1.05	25.05 ± 1.09
	Eyes with abnormal corneal morphology	25.02 ± 1.17	25.01 ± 1.16
	Eyes after corneal refractive surgery	24.99 ± 1.02	25.00 ± 1.03
	Cataract eyes	23.17 ± 0.49	23.12 ± 0.52
	Eyes after cataract surgery	23.17 ± 0.43	23.22 ± 0.45
Statistical analysis	Mean of all groups	24.28 ± 1.25	24.29 ± 1.26
	95CI% of the mean of the difference	-0.01 ± 0.06(-0.02, 0.01)	
	Cutoff value VS. 95%CI	Difference VS. -0.15	Difference VS. 0.15
	T test	18.84	-21.34
	P value	< 0.001	< 0.001

## Discussion

The OCT is usually an important auxiliary imaging equipment for the diagnosis of anterior segment diseases. Central corneal thickness measurement is of great significance for refractive surgery screening and the diagnosis of keratoconus [21]. The IOL Master 500 is commonly used for ocular biology measurement. Moreover, measuring axial length plays an essential role in evaluating myopia progression [22]. The AOCT-1000 M can measure both central corneal thickness and axial length. Its principles for corneal imaging, and axial length measurement are similar to RTVue XR, and IOL Master 500 respectively. Therefore, these two devices were selected as control devices to verify the repeatability and agreement of their measurements.

The repeatability of the instrument is one of the important indexes to judge the measurement reliability. In previous studies, the central corneal thickness repetition coefficient ICC of RTVue was higher than 0.992 [20, 23–26], with high repeatability. In this study, the ICC of the central corneal thickness of RTVue XR is 0.998, and AOCT-1000 M is 0.994, which indicate that the retest accuracy of the central corneal thickness is high. Typically, ICC is greater than 0.75 [27] represents a good repeatability. In this study, the ICC of 2–5 mm and 5–6 mm around AOCT-1000 M are all higher than 0.75, which indicate that the measurement of corneal thickness around the two devices is also reliable, but the repeatability is lower compared with the central corneal thickness.

In the past research on the agreement of central corneal thickness measurement between RTVue and other devices, RTVue showed poor agreement with Sirius, AL Scan, Galilei, USP and other devices [19, 24, 28], which could not be replaced. There were some differences in measurement principles or image processing methods among various devices. However, the corneal thickness measurements of RTVue XR and Cirrus-5000 [29] had small difference and high agreement, which could be reliable to use. In this study, the principle of AOCT-1000 M was similar to that of RTVue XR. The average central corneal thickness values of 58 eyes measured by the two devices were 504.46 ± 42.53 μm and 504.43 ± 42.89 μm respectively. The average difference between groups was 0.03 μm, and the 95% confidence interval is (-1.17, 1.24), which was much smaller than the set threshold value of 15 μm(p < 0.001). The Bland-Altman diagram showed that the 95% consistency interval between them was (-8.88, 8.95), which was also within the set boundary value. And for different types of diseases (group), including keratoconus, postoperative corneal refractive surgery, cataract, postoperative cataract surgery and normal eyes, all showed small difference. Therefore, the measurement of central corneal thickness could be replaced by two devices. As the measurement moved to the periphery, the difference between the two groups became larger. In the range of 2–5 mm, the maximum value of the 95% confidence interval of the difference between the two groups in eight directions was 2.23 μm, and the maximum value of the lower limit was 7.21. Bland-Altman showed that the maximum value of the 95% LOA of the two groups was 19.49 μm and the maximum value of the lower limit was − 24.79 μm. Within the range of 5–6 mm, the maximum value of the 95% confidence interval of the difference between the two groups was 10.71 μm and the maximum value of the lower limit was − 13.62 μm. And its corresponding Bland-Altman showed that the maximum value of the 95% LOA of the two groups was 48.64 μm and the maximum value of the lower limit was − 45.88 μm. From the widening range of the maximum upper limit and the maximum lower limit of the difference, as well as the decrease of ICC of the two devices, it could be seen that the measurement difference of the instruments would increase further to the periphery. It might be affected by the eyelids and eyelashes. But on the other hand, since the subjects examined on different devices, their hand positions were slightly different, so the boundary of different devices in the central and peripheral corneas might not be completely coincident. Therefore, in clinical work, it was more reasonable to choose the same equipment if the thickness of the peripheral cornea of the same subject was compared longitudinally. If conditions do not allow, when using different equipment, knowing the approximate difference range can also provide a certain reference for the comparison between the measured values of different equipment.

IOL master 500 is relatively fast, convenient and non-invasive. The repetition coefficient ICC could reach 1.000 [11, 12], the repetition coefficient was very high. In this study, the ICC of IOL Master and AOCT-1000 M was 1.000, which was consistent with previous studies. The repeatability and stability of the equipment were good, which could provide a reliable tool for clinical diagnosis and treatment.

In addition to IOL master 500, there were SW9000, LS 900, A-scan and other devices in clinical measurement. It was found that the differences between IOL master and the above devices were between 0.002 and 0.2 mm [15–18]. In this study, the difference of axial length of 58 eyes measured by IOL Master 500 and AOCT-1000 M was − 0.01 on average, and the 95% confidence interval is between (-0.02, -0.01), which was significantly smaller than the boundary value of 0.15. At the same time, the 95% consistency interval between them in the Bland-Altman diagram was (-0.11, 0.1), which was also within the set range. It showed excellent agreement between the two devices, and they could be used as substitutes in clinical axial length measurement.

## Conclusion

In summary, AOCT-1000 M has good repeatability in measuring central corneal thickness and axial length, further demonstrating high agreement with RTVue XR and IOL Master 500, so they are interchangeable. The actual imaging range of AOCT-1000 M can reach 20 mm, and it can image the anterior segment including sclera. It can be applied to contact lenses fitting, including OK lens, soft lens and scleral lens. The AOCT-1000 M possesses the capability to measure the tear thickness under the lens. At the same time, the axial length can be accurately measured. It has a strong clinical application potential for some ophthalmology and optometry clinics which intend to control the cost, reduce the floor space and obtain accurate eye parameter data.

## Data Availability

Not applicable.
